# The influence of warm ischemia elimination on kidney injury during transplantation – clinical and molecular study

**DOI:** 10.1038/srep36118

**Published:** 2016-11-03

**Authors:** Dorota Kamińska, Katarzyna Kościelska-Kasprzak, Paweł Chudoba, Agnieszka Hałoń, Oktawia Mazanowska, Agnieszka Gomółkiewicz, Piotr Dzięgiel, Dominika Drulis-Fajdasz, Marta Myszka, Agnieszka Lepiesza, Wojciech Polak, Maria Boratyńska, Marian Klinger

**Affiliations:** 1 Department of Nephrology and Transplantation Medicine, Wroclaw Medical University, Wrocław 50-556, Poland; 2 Department of General, Vascular and Transplant Surgery, Wroclaw Medical University, Wrocław 50-556, Poland; 3 Department of Pathomorphology and Oncological Cytology, Wroclaw Medical University, Wrocław 50-556, Poland; 4 Faculty of Medicine and Dentistry, Wroclaw Medical University, Wrocław 50-556, Poland; 5 Department of Histology and Embryology, Wroclaw Medical University, 50-368, Wrocław, Poland; 6 Department of Surgery, Division of HPB and Transplantation Surgery, Erasmus MC, University Medical Center Rotterdam, Rotterdam, the Netherlands

## Abstract

Kidney surface cooling was used during implantation to assess the effect of warm ischemia elimination on allograft function, histological changes and immune-related gene expression. 23 recipients were randomly assigned to a group operated on with kidney surface cooling during implantation (ice bag technique, IBT group), and the other 23 recipients receiving the contralateral kidney from the same donor were operated on with a standard technique. Three consecutive kidney core biopsies were obtained during the transplantation procedure: after organ recovery, after cold ischemia and after reperfusion. Gene expression levels were determined using low-density arrays (Format 32, TaqMan). The IBT group showed a significantly lower rate of detrimental events (delayed graft function and/or acute rejection, p = 0.015) as well as higher glomerular filtration rate on day 14 (p = 0.026). A greater decrease of *MMP9* and *LCN2* gene expression was seen in the IBT group during total ischemia (p = 0.003 and p = 0.018). Elimination of second warm ischemia reduced the number of detrimental events after kidney transplantation, and thus had influence on the short-term but not long-term allograft function. Surface cooling of the kidney during vascular anastomosis may reduce some detrimental effects of immune activation resulting from both brain death and ischemia-reperfusion injury.

The quality of organs removed from deceased donors clearly is one of the most crucial factors in determining graft survival and function of a kidney transplant. The most deleterious effect was described for donor age^1^, while the length and type of ischemia seem to be crucial after organ retrieval. Cold ischemia time (CIT) has been widely reported to affect renal allograft survival and function^2^. During organ procurement the gradual warming increases cell metabolism, accelerating detrimental changes in the transplanted allograft. Warm ischemia time (WIT) is a period which begins at the time of removal of the procured organ from storage ice and ends with the initiation of graft reperfusion, depending on the anastomosis time of renal vessels. Prolongation of WIT is not only detrimental for immediate renal allograft function but also influences long-term results^3^,^4^. Prolonged WIT was also associated with longer hospitalization after kidney transplantation^5^ and impaired long-term graft survival after living donation^6^ as well as deceased donor^7^ kidney transplantation.

It was postulated that maintaining a lower kidney core temperature during implantation may reduce kidney allograft injury. Since the 1980s several attempts have been made to achieve kidney cooling during anastomosis. However, none of the previous studies (all of which were small, single-center retrospective and uncontrolled studies) noted a significant improvement in the posttransplant graft function.

The aim of this randomized study was to assess whether elimination of warm ischemia by graft surface cooling during implantation improves kidney allograft outcome. We also wanted to analyze allograft histological changes as well as altered gene expression of factors involved in the immune response in relation to kidney transplantation outcome. The trial was registered in the ISRCTN registry and was provided with the following reference number: ISRCTN10879277 (15Jun2016).

## Results

### Study procedure

In this study the analysis was performed on matched pairs of kidneys, which means that two kidneys were harvested from the same donor and one of them was transplanted into a recipient using kidney surface cooling during implantation (ice bag technique, IBT group), and the other using a standard technique (ST group).

The kidney was cooled in a specially designed disposable polyethylene bag. Three consecutive kidney core biopsies were obtained during the transplantation procedure for pathological examination, immunohistochemistry and gene expression analysis:

- biopsy 1 (B1): just after organ recovery,
- biopsy 2 (B2): after organ storage and cold ischemia,
- biopsy 3 (B3): about 30 min after reperfusion.

### Clinical data

The kidneys were transplanted after cold ischemia time (CIT) ranging from 12 to 44 hours. Warm ischemia time in the ST group ranged from 15 to 44 minutes.

The initial immunosuppressive therapy consisted of tacrolimus, mycophenolate mofetil/sodium and prednisone in 29 cases; cyclosporin A, mycophenolate mofetil/sodium and prednisone in 16 cases; one recipient was treated with tacrolimus, everolimus and prednisone. Three recipients received induction therapy with mono- or polyclonal antibodies. The details of the transplantation procedure as regards HLA matching, CIT and immunosuppressive therapy did not differ between the studied groups.

Kidney recipients were followed for five years. The recipients from the two examined groups were similar as regards clinical pre-transplant factors (age, gender, immunization, reason of chronic kidney disease, type and length of dialysis). As for peri- and posttransplant factors (CIT, immunosuppression) only the warm ischemia time (WIT) differed between the groups.

Eight recipients from the ST group and five from the IBT group developed delayed graft function (DGF, defined as the use of dialysis within seven days of the transplant). During the first posttransplant year, biopsy-proven acute rejection episodes (BPAR, including borderline-“suspicious” for acute T-cell-mediated rejection) were observed in 10 recipients from the ST group in contrast to only four recipients from the IBT group. Anti-rejection treatment consisted of pulses of steroids in all cases, with thymoglobuline in four cases of antibody-mediated rejection (AMR). In all cases of acute rejection complete remission after treatment was recorded. The average time of hospital stay did not differ between the groups. Taking into consideration all posttransplant immune-related pathologies (DGF and/or BPAR) in individual recipients, we found that the IBT group had a significantly lower rate of detrimental events compared to the other group (p = 0.015). Data are shown in Table 1. No transplant site infection was observed in either of the groups. The use of IBT device was not connected to the increase of infection on transplantation site.

Four recipients died during the observation period with the functioning graft – two from the ST group (one recipient died two months after transplantation due to a generalized infection and the other one died two years after the transplantation due to aortic aneurysm) and two from the IBT group (three months after transplantation due to non-compliance and one month after transplantation due to *Clostridium difficile* infection). None of the patients had to recommence dialysis. After five-year observation period data were not available for 42 study participants (91%).

The allograft function was assessed by estimated GFR (according to the Modification of Diet in Renal Disease, MDRD formula). Elimination of WIT was associated with significantly better early allograft function. The groups differed only in the early posttransplant period with statistical significance on day 14 in favor of the IBT group (Table 2).

### Histological changes

The histological assessment of biopsies included the Remuzzi score (Table 3)^8^. Biopsies 1 presented relatively high Remuzzi score, probably due to high incidence of atheromatous changes in donors, most of whom died due to cerebrovascular accident. The three biopsies did not differ significantly as regards the main histological features (glomerular sclerosis, tubular atrophy, interstitial fibrosis and arterial narrowing). We observed that ischemia was followed by vacuolization and necrosis of proximal tubular epithelial cells as well as interstitial edema. The changes were described by the TOV index (T – tubular necrosis, O – interstitial edema, V – tubular cell vacuolization) and graded 0–3 (0 – none, 1 – low grade, 2 – mild, 3 – high grade). None of the biopsies presented grade 3 of the TOV index (Table 4). The glomerular changes in postischemic biopsies were generally unremarkable. The postreperfusion biopsies did not differ between ST and IBT groups.

To obtain a picture of the key chronic pathological processes involving kidney tissue in the immunohistochemical analysis, we assessed alpha-SMA as a marker of tissue fibrosis and two pro-apoptotic molecules: p53 and Bcl-2. The staining was assessed according to the Remmele immunoreactive score – IRS (0 to 12)^9^. The groups did not differ with regard to alpha-SMA, p53 and Bcl-2 protein expression at any time point (B1, B2, B3) in either tubular or glomerular segments (Table 5).

### Gene expression

**Figure 1:**
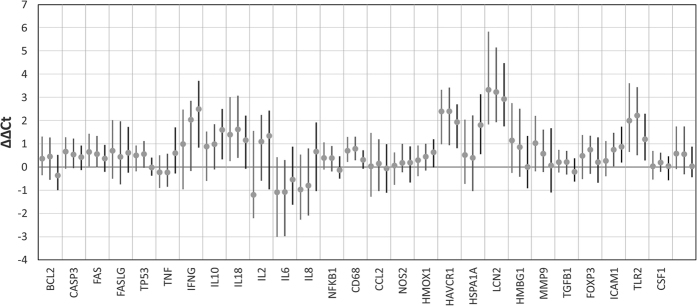
Gene expression presented as ΔΔCt (dot-median, line-interquartile range). Data presented for each of the three biopsies (B1-light gray, B2-dark grey, B3-black).

We analyzed the expression of 29 genes involved in key peritransplant immune reactions as follows:

- tissue injury: *CSF1*, *IL8*, *NFKB1*, TGFB1, *MMP9*, *HSPA1A*, *HMOX1*, *NOS2*, *LCN2*, *HAVRC1*, *HMGB1*, *TLR2*, *IL17A* and *GUSB*
- inflammation: *IL6*, *IL10*, *TNF*
- T-cell activation: *IL2*, *IFNG*, *FAS*, *FASLG*
- cell migration: *ICAM1*, *CCL2*, *CD68*
- apoptosis: *BCL2*, *TP53*, *CASP3*, *IL18*.
- Gene expression data are presented in Fig. 1.

#### Posttransplant events

The increased expression of apoptosis-related gene *CASP3* (p = 0.035) and markers of kidney injury *TLR2* (p = 0.030) and *GUSB* (p = 0.049), and decreased expression of *HMOX* (p = 0.047) during cold ischemia (B1 → B2) correlated positively with acute rejection episodes.

High expression of matrix metallopeptidase 9 (*MMP9*) and neutrophil gelatinase-associated lipocalin (NGAL; *LCN2*) genes in postreperfusion biopsy (B3) correlated with the presence of DGF (p = 0.012 and p = 0.032, respectively). Increase of *IL10* gene expression during cold ischemia (B1 → B2) as well as *ICAM* at the same time was related to the presence of DGF (p = 0.032 and 0.049, respectively).

The recipients who developed DGF and/or BPAR (event-positive group) presented two times higher MMP9 expression after reperfusion (B3, p = 0.009).

Gene expression for the glycoprotein kidney injury molecule 1 (KIM-1) *HAVCR* in the second biopsy (B2) as well as the level of increase during cold ischemia (B2 → B1) were higher in the event-positive group (p = 0.048 and p = 0.025, respectively). A similar association was observed for *ICAM* gene expression (p = 0.012 and p = 0.003, respectively). The event-positive group also showed increased *TNF* gene expression (B2, p = 0.049) and increase of *HMOX1* gene expression during cold ischemia (B2 → B1; p = 0.024).

### Univariate analysis

#### Early allograft function

Early allograft function was associated with the expression of *LCN2*, *MMP9*, *HMOX* and *ICAM1.*

*LNC2* gene expression correlated negatively with posttransplant kidney allograft function described as eGFR on day 14 and at 1 and 3 months (B3, 14 days: rs = −0.24, p = 0.037; 1 month: rs = −0.34, p = 0.004; 3 months: rs = −0.29, p = 0.033). Lower postreperfusion expression of *MMP9* gene was related to better short-term graft function up to one month (B3, 14 days: rs = −0.26, p = 0.024; 1 month: rs = −0.25, p = 0.040). Higher *HMOX1* expression after reperfusion was associated with worse short-term function (B3, 14 days: rs = −0.25, p = 0.024; 1 month: rs = −0.24, p = 0.037). Higher final expression for *ICAM* was linked to worse short-term graft function (B3, 14 days: rs = −0.230, p = 0.040; 1 month: rs = −0.267, p = 0.020; B3 → B1, 14 days: rs = −0.298, p = 0.007).

#### Long-term allograft function

Allograft function between three months and two years was associated with apoptosis-related gene expression (*BCL2*, *FAS*, *TP53*, *IL18*) and *TGFB*.

The reduced expression of apoptosis-related gene *BCL2* during ischemia was linked to better graft function at 3 and 18 months (B3 → B1, 3 months: rs = −0.278, p = 0.037; 18 months: rs = −0.313, p = 0.035; B3 → B2, 18 months: rs = −0.317, p = 0.038). *FAS* gene expression decrease during total ischemia as well as during warm ischemia was associated with better allograft function after 18 months (B3 → B1: rs = −0.395, p = 0.007; B3 → B2: rs = −0.332, p = 0.029). Reduction of the other apoptosis determinants *TP53* and *IL18* during ischemia time was associated with better long-term allograft function up to 24 months (*TP53*, B3 → B2, 12 month: rs = −0.324, p = 0.022; 18 months: rs = −0.442, p = 0.003; 24 months: rs = −0.351, p = 0.022; *IL18*, B3 → B1, 18 months: rs = −0.351, p = 0.017; 24 months: rs = −0.326, p = 0.028).

A higher decrease of *TGFB* gene expression during warm ischemia was seen in grafts with better function from 3 to 24 months (B3 → B2, 3 months: rs = −0.323, p = 0.017; 6 months: rs = −0.394, p = 0.004; 12 months: rs = −0.387, p = 0.005; 18 months: rs = −0.444, p = 0.002; 24 months: rs = −0.354, p = 0.019).

### Multivariate analysis

We analyzed key detrimental factors and their influence on posttransplant outcome (CIT, WIT, donor age, recipient age, gene expression). Only WIT (p = 0.044) and donor age (p = 0.009) were independent predictors of AR and/or DGF. None of the molecular markers proved to be an independent risk factor for worse posttransplant outcome.

### The analysis was performed on matched pairs of kidneys

**Figure 2:**
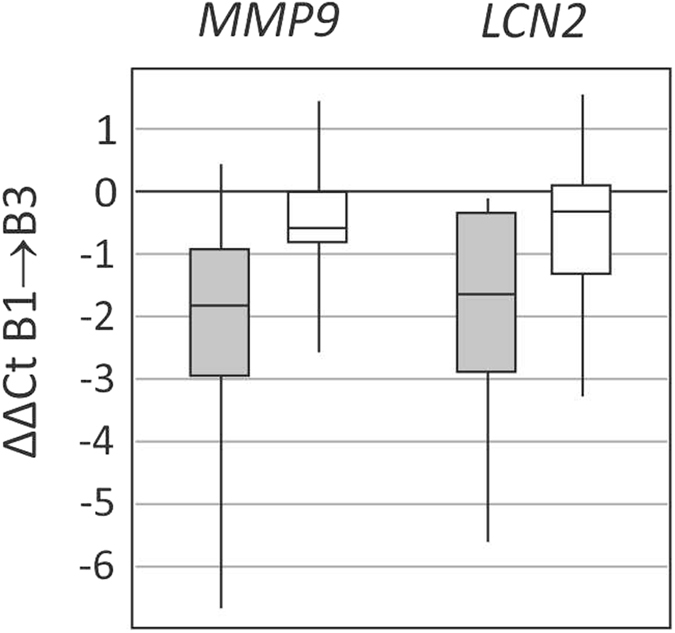
The change in *MMP9* and *LCN2* gene expression levels over ischemia time in matched pairs of the kidneys from the same donor as observed in IBT (grey box) vs ST group (white box). Data presented as median (horizontal line), interquartile range (box), and values range (vertical line).

The analysis performed on matched pairs of kidneys (two kidneys from the same donor, one operation using standard technique and the other operation with elimination of warm ischemia) showed no differences in gene expression except for *MMP9* and *LCN2.* During total ischemia the ST group showed no change in *MMP9* expression level, whereas in the IBT group the expression decreased almost threefold (B3 → B1, p = 0.003). Similarly, the IBT group showed a greater reduction of *LCN2* gene expression during total ischemia (B3 → B1, threefold, p = 0.018) (Fig. 2).

## Discussion

This is the first randomized study showing that lower kidney core temperature during graft implantation reduces injury to the kidney allograft and influences its postoperative function as well as the rate of immune-related detrimental posttransplant events i.e. delayed graft function and acute rejection episodes. In this study we present data confirming elimination of the deleterious effect of warm ischemia time on kidney allograft function as well as immune-related allograft gene expression. To the best of our knowledge, this is also the first study showing the effect of elimination of warm ischemia time on intragraft gene expression, indicating that graft surface cooling may mitigate the previously activated immune response within the transplanted kidney.

The quality of the deceased donor organ depends on donor-related factors, such as donor age, gender, body mass index, kidney weight/volume and donor comorbidity^10^. Donor brain death as well as ischemia-reperfusion injury are known to influence kidney allograft function. As regards peritransplant factors, cold ischemia time causes delayed graft function and may impair graft survival^11^. The mean CIT varies among centers but most often it is between 10 and 18 hours^7^. In our study, the mean CIT was 26 hours since in Poland the recipient typically travels to the kidney^12^, and was longer than 24 hours in 74% of recipients. We did not find an effect of CIT prolongation on allograft function and examined gene expression. The effect may be so pronounced that further changes in the duration of ischemia are negligible when CIT is much longer than 24 hours.

Warm ischemia time, also called anastomosis time (WIT), is another potentially modifiable factor in kidney transplantation outcome. It depends on both the technical skills of the transplant surgeon and procedure conditions – vessel number and anatomical anomalies of graft, recipient’s obesity, etc.

The previous studies reported that WIT triggers injury primarily to proximal tubular cells^13^ and may induce acute tubular necrosis which leads to delayed graft function. However, the results of clinical studies analyzing the importance of the WIT are indecisive^14^.

A few authors have reported that shorter WIT was associated with decreased incidence of acute tubular necrosis after renal transplantation^15^, but others found no significant effect of first and second WIT on the risk of renal graft failure^16^,^17^,^18^. Measurement of the kidney core temperature may elucidate the different findings^15^,^19^. Some data indicated that the clinical relevance of WIT shorter than approximately 30 min was minimal^20^.

Recently, two big studies analyzing the importance of warm ischemia in kidney transplantation were published. Tennankore *et al*. in a study on 131,677 kidney transplant recipients found that WIT longer than 30 min was associated with a statistically higher adjusted relative hazard for the composite event of death or graft failure. In the case of WIT > 60 min, a 23% increase in the adjusted relative hazard for death or graft failure was observed^4^. Similarly, Heylen *et al*. reported the effect of anastomosis time on allograft outcome in 669 first single kidney transplantations. WIT independently increased the risk of delayed graft function and independently impaired allograft function after transplantation. In the subsequent protocol biopsies, prolonged WIT was associated with increased risk of interstitial fibrosis and tubular atrophy^3^. Also Weissenbacher in a study on 1245 consecutive deceased donor kidney transplantations in Europe found that anastomosis time was an independent significant factor not only for graft but also for patient survival^7^. Prolonged WIT was also linked to longer hospitalization after kidney transplantation. Every five min of longer anastomosis time was associated with one extra day in hospital due to delayed graft function^1^,^5^. A deleterious influence of prolonged warm ischemia on long-term graft survival was also observed after living donor transplantation^6^. Most of the studies indicated that WIT longer than 30 min should be considered as a major, potentially modifiable risk factor for inferior long-term results after kidney transplantation.

During anastomosis time, kidney temperature increases logarithmically at a rate of 0.48 °C/min, reaching 26.7 °C at the beginning of reperfusion^19^. It was shown that kidney core temperature rose significantly during vessel anastomosis, and an increase above 8 °C was associated with delayed graft function^21^. Maintaining the temperature of the kidney during the time of vascular anastomosis below 16 or 17 °C decreased the risk of acute tubular necrosis^15^.

Several attempts have been made to achieve kidney cooling during anastomosis, from the ice-filled jacket^22^, stockinette^23^ and holding net with attached cold drip infusion set^15^ in the past, to the polyethylene bags filled with ice (IBT)^21^,^24^,^25^,^26^,^27^ or retroperitoneal cooling during robotic procedures^28^ at present time. In our study, cooling was achieved using a specially designed polyethylene bag filled with slushed sterile ice in which the kidney was placed until reperfusion. This technique allowed us to maintain a stable temperature of +4 °C for the transplanted kidney. The IBT was feasible, and we did not observe any technique-related complications. The previous studies, while informative, failed to demonstrate the influence of IBT on subsequent graft function and transplant outcome^24^,^26^. Thanks to the paired-kidney randomized analysis concerning grafts from the same donor, we were the first to show the correlation between effective cooling and not only short-term allograft function but also reduced number of posttransplant detrimental events like acute rejection and delayed graft function^29^. The effect was so evident that it was statistically significant despite the relatively small study group.

The pathological processes after donor brain death and cold/warm ischemia are observed not only at the functional level but also in the evoked histological changes as well as altered gene expression^30^,^31^,^32^.

To provide insight into time-dependent intragraft changes, we performed three sequential biopsies during the transplantation procedure. We assessed alpha-SMA as a marker of tissue fibrosis and two apoptosis-related molecules: p53 and Bcl-2. In our study we did not observe any influence of warm ischemia elimination on graft histological changes as well as immunohistochemical analysis. The lack of histological changes or protein expression alterations in our study was probably caused by the relatively short WIT (approx. 23 min.) Further analysis looked beyond the structural changes to include gene expression assessment.

It was previously reported that donor brain death caused activation of stress-related response with some immune-related gene activation^33^,^34^. Gene expression can be amplified by the detrimental effects of cold ischemia^35^,^36^,^37^ and warm ischemia^30^,^32^,^38^,^39^.

We have previously reported that in procurement biopsies, as well as in those taken after cold ischemia and reperfusion, cytokine gene expression was even higher than during the period of acute rejection^40^. Saat *et al*. observed in animal model that donor brain death is responsible for the majority of gene expression induction with no changes during cold ischemia and only mild changes after warm ischemia time^41^. Similarly, in our study we have observed major changes after donor brain death with no influence of cold ischemia time on most of the genes expression even over longer time (mean CIT about 27 hours) with the exception of the decrease of MMP9 and LCN2 expression.

The altered gene expression was reported to influence histological parameters of kidney biopsies^42^ as well as to affect kidney function and graft survival^32^,^43^.

In our study we found that high gene expression of the kidney injury markers: matrix metallopeptidase 9 and NGAL after reperfusion biopsy was associated with the presence of DGF and deteriorated short-term allograft function up to three months. Moreover, recipients who presented a complicated posttransplant course with a delayed graft function and/or acute rejection showed two times higher *MMP9* expression after reperfusion. This was an expected finding, because the two molecules have previously been reported as markers of acute kidney injury^44^.

Allograft function between three months and two years was associated with apoptosis-related gene expression (*BCL2*, *FAS*, *TP53*, *IL18*) and expression of the fibrosis-related gene *TGFB*. The level of decreased activation of the above genes during ischemia was linked to better long-term graft function up to 24 months. A similar observation of Goncalves *et al*. indicated that a higher Bax/BCL2 ratio in preimplantation biopsies as well as higher *TGFB* expression were linked to a higher incidence of DGF^45^. Also in our previous study performed on a different group of kidney recipients we have reported that increased expression of *CASP3* and *TP53* was related with higher incidence of delayed graft function^46^. In that study we reported that the level of apoptosis-gene expression was strongly dependent on CIT.

Our analysis performed on matched pairs of kidneys from the same donor allowed us to eliminate the potential effect of donor-related factors on the study results and to assess only the effect of WIT elimination itself. We found that gene expression of previously activated kidney injury markers (*MMP9* and *LCN2*) remained almost unchanged during warm ischemia time, whereas IBT was associated with almost threefold decrease of *MMP9* and *LCN2* gene expression. It suggests that IBT may effectively eliminate the detrimental influence of warm ischemia, also on the molecular level. To our knowledge, no such studies have been published before.

## Conclusion

From a clinical perspective, elimination of warm ischemia time exerted a positive impact exclusively on early events and significantly diminished the delayed graft function and rejection rate and influenced short-term allograft function. In long-term observation there were no differences in allograft function up to 5 years. It seems reasonable to expect that the beneficial clinical effect could be more pronounced if the warm ischemia elimination approach was applied to extended criteria donors.

Increased gene expression in kidney biopsies retrieved from deceased donors is further augmented during cold and warm ischemia and reperfusion. The kidney surface cooling during vascular anastomosis may reduce some detrimental effects of immune activation resulting from both brain death and ischemia-reperfusion injury.

## Material and Methods

46 kidney allograft recipients (17 females and 29 males aged between 21 and 71 years) who underwent transplantations at Wroclaw Medical University between 2006 and 2009 were enrolled in this study. They received grafts from 23 deceased donors (10 females and 13 males aged between 20 and 63 years). There were no living or donation-after-circulatory-death recipients. All kidney allograft recipients were randomly assigned to one of two groups before transplantation. The randomization was performed when the first recipient from each pair was admitted for transplantation; at the moment of enrollment to the study he/she was randomized to one of the two study groups (IBT or ST). Half of the recipients were operated on with kidney surface cooling during the time of implantation (ice bag technique, IBT group), and the other recipients receiving the contralateral kidney from the same donor were operated on with a standard technique (ST group). In 10 cases out of 23 pairs (44%), the first recipient was operated using IBT.

The donor and recipient detailed characteristics are presented in Table 6. The examined groups did not differ significantly as regards the pre-transplant features.

The cooling was achieved using a specially designed disposable polyethylene bag produced by Raguse GmbH (Germany). The kidneys were prepared for implantation on the back table and put into a polyethylene bag in order to eliminate warm ischemia. The bag consisted of three compartments. The middle compartment homing the graft was surrounded by two external compartments filled with ice-cooled sterile saline and placed on the melting slushed ice. After placing the graft in the middle compartment, the renal vein and artery were pulled outside the bag via a small hole in the middle compartment. Then the vascular renal vein and renal artery were anastomosed to the iliac vein and artery. After completion of the vascular anastomoses followed by graft reperfusion, the polyethylene bag was removed from the transplanted kidney.

Three consecutive kidney core biopsies were obtained with a 16 G automatic needle (TSK Laboratory, Japan) during the transplantation procedure:

- biopsy 1 (B1): just after organ recovery,
- biopsy 2 (B2): after organ storage and cold ischemia, on the back table, before the transplantation procedure,
- biopsy 3 (B3): about 30 min after reperfusion.

The inner part of the kidney core biopsies (cortex + medulla), which was subjected to standard histopathological assessment (according to Banff’05 criteria) and immunohistochemical analysis, was stored in buffered formalin. The outer part (cortex) used for gene expression studies was placed in RNAlater stabilization solution (Qiagen, Hilden, Germany) and stored at −70 °C.

Gene expression levels were determined using low-density arrays (Format 32, TaqMan). Three reference genes were studied: *ACTB* (Hs99999903_m1), *GAPDH* (Hs99999905_m1), *18S* (Hs99999901_s1). *GAPDH* was chosen as a reference gene for its lowest cycle threshold (Ct) variability between studied kidney samples from the three stages of the procedure.

10 kidney biopsies obtained from living donors were chosen as a reference group (ref) for data normalization. The expression data are presented as ΔΔCt = mean ∆Ct_ref_ − ∆Ct_sample_, where ∆Ct = Ct_gene_ − Ct_GAPDH_, and Ct is the cycle threshold value and defines the calculated cycle number in which the fluorescence measured during the PCR reaction increases over the preset threshold value. Also the change in expression level during the transplantation procedure (from biopsy *n* to biopsy *m*) is described as ΔΔCt = ∆Ct_biopsy m_ − ∆Ct_biopsy n_.

We examined the expression of 29 genes involved in various aspects of the alloimmune response, i.e. tissue injury, inflammation, T-cell activation, cell migration and apoptosis:

*BCL2* (Hs00608023_m1), *CASP3* (Hs00263337_m1), *FAS* (Hs00236330_m1), *FASLG* (Hs00181225_m1), *TP53* (Hs00153349_m1), *TNF* (Hs00174128_m1), *IFNG* (Hs00174143_m1), *IL10* (Hs00174086_m1), *IL18* (Hs00155517_m1), *IL2* (Hs00174114_m1), *IL6* (Hs00174131_m1), *IL8* (Hs00174103_m1), *NFKB1* (Hs00765730_m1), *CD68* (Hs00154355_m1), *CCL2* (Hs00234140_m1), *NOS2* (Hs00167248_m1), *HMOX1* (Hs00157965_m1), *HAVCR1* (Hs00273334_m1), *HSPA1A* (Hs00359163_s1), *LCN2* (Hs00194353_m1), *HMGB1* (Hs01923466_g1), IL17A (Hs99999082_m1), *MMP9* (Hs00234579_m1), *TGFB1* (Hs99999918_m1), *FOXP3* (Hs00203958_m1), *ICAM1* (Hs00164932_m1), *TLR2* (Hs01872448_s1), *CSF1* (Hs00174164_m1), *GUSB* (Hs99999908_m1).

Standard histological assessment as well as immunohistochemical analysis were performed on paraffin-embedded sections with avidin-biotin-peroxidase complex. The monoclonal antibodies used were p53 (Monoclonal Mouse Anti-Human p53 Protein, Clone DO-7, No. M7001, DAKO), Bcl-2 (Monoclonal Mouse Anti-Human BCL2 Oncoprotein, No. M0887, DAKO), and α-SMA (Mouse Anti-Human Alpha-Smooth Muscle Actin Monoclonal Antibody, No. MAB1420, R&D Systems). Results were presented as IRS (Immunoreactive Remmele Score)^9^.

### Statistical analysis

Statistical analysis was performed using the Statistica v.12 package (Statsoft, Poland). For continuous variables, Mann-Whitney U-test and Spearman correlation were used. Paired analysis was performed with Wilcoxon signed-rank test. The Chi-square test or Fisher’s exact test, if data were sparse, were used to compare categorical variables. Multivariate analysis was performed with logistic regression. p value < 0.05 was considered significant. Statistical analysis was performed on matched pairs of kidneys – two kidneys from the same donor, one operation using standard technique and the other operation with warm ischemia eliminated. The study design allowed us to eliminate the potential effect of donor-related factors.

The study was approved by the Wroclaw Medical University Bioethics Committee, in accordance with the World Medical Association’s Declaration of Helsinki. Each patient had read the information sheet and provided their fully informed consent. All methods were performed in accordance with the relevant guidelines and regulations.

## Additional Information

**How to cite this article**: Kamińska, D. *et al*. The influence of warm ischemia elimination on kidney injury during transplantation – clinical and molecular study. *Sci. Rep.* **6**, 36118; doi: 10.1038/srep36118 (2016).

**Publisher’s note**: Springer Nature remains neutral with regard to jurisdictional claims in published maps and institutional affiliations.

## Figures and Tables

**Table 1:** Distribution of detrimental posttransplant events between studied groups.

	STANDARD	IBT	p
**Time of hospitalization (days)**	26.6 ± 9.8	22.4 ± 11.8	0.82
**DGF**	8 (35%)	5 (22%)	0.51
**Total BPAR**	10 (43%)	4 (17%)	0.11
**AMR**	2 (8%)	2 (8%)	1.00
**T-cell mediated rejection (borderline/IA,B/IIA)**	2/4/2	1/0/1	0.074
**EVENT (BPAR and /or DGF)**	14 (61%)	6 (26%)	**p** = **0.015**

*DGF – delayed graft function, BPAR – biopsy-proven acute rejection in 1^st^ year, AMR – antibody-mediated rejection, assessment according to Banff’05 criteria.

**Table 2:** Kidney allograft function according to type of transplant procedure (ST vs IBT, paired analysis).

	STANDARD		IBT		p
	mean ± SD	median	mean ± SD	median	
GFR 7 days	24 ± 18	25	38 ± 22	38	0.065
GFR 14 days	31 ± 18	34	43 ± 19	43	**0.026**
GFR 1 month	40 ± 19	36	45 ± 18	50	0.10
GFR 3 months	47 ± 15	45	44 ± 21	38	0.63
GFR 6 months	46 ± 12	42	47 ± 14	44	0.92
GFR 12 months	49 ± 18	45	49 ± 17	46	0.59
GFR 18 months	52 ± 12	48	50 ± 15	50	1.00
GFR 24 months	48 ± 16	45	52 ± 19	50	0.50
GFR 60 months	47 ± 19	50	49 ± 21	49	0.55

**Table 3:** Histological evaluation of biopsy 1 with Remuzzi score (p = ns).

	ST	IBT
	Mean [values]	
**Glomerular sclerosis**	0.7 [0–1]	0.6 [0–1]
**Tubular atrophy**	0.7 [0–1]	0.8 [0–1]
**Interstitial fibrosis**	0.2 [0–1]	0.3 [0–1]
**Arterial and arteriolar narrowing**	0.8 [0–2]	0.7 [0–1]
	(Mean ± SD)	
**Remuzzi score** (mean ± SD)	2.6 ± 1.6	2.4 ± 1.5

*Remuzzi score ST vs IBT p = ns.

**Table 4:** Histopathological assessment of biopsies – tubular compartment (TOV index); T – tubular necrosis, O – edema of epithelial cells, V – vacuolization of epithelial cells.

TOV*	Biopsy 1 (ST/IBT)			Biopsy 2 (ST/IBT)			Biopsy 3A ST			Biopsy 3B IBT			p 3A vs 3B
	0	1	2	0	1	2	0	1	2	0	1	2	ns
**T**	22/22	1/1	0/0	9/7	11/9	5/5	8	23	15	12	23	11	ns
**O**	17/21	4/2	1/1	7/11	10/14	1/3	13	26	7	16	25	5	ns
**V**	18/16	3/5	1/3	7/6	11/12	6/4	8	26	12	8	26	12	ns

^*^TOV index: 0 – no changes, 1 – low grade, 2 – mild, 3 – high grade (high grade was absent in all biopsies).

**Table 5:** Immunohistochemical analysis of kidney tissue.

	Biopsy 1 (ST/IBT)			Biopsy 2 (ST/IBT)			Biopsy 3A ST			Biopsy 3B IBT			p 3A vs 3B
	0	1	2	0	1	2	0	1	2	0	1	2	
alpha-SMA g	13/19	0/0	2/3	14/12	2/0	1/0	6	6	0	7	7	1	ns
alpha-SMA t	5/7	10/9	4/3	3/2	7/7	10/9	1	4	14	1	3	13	ns
p53 g	16/13	1/2	0/0	12/16	0/1	1/0	10	2	0	13	2	0	ns
p53 t	5/5	12/10	3/2	1/2	10/13	6/5	1	8	9	2	9	6	ns
Bcl-2 g	4/6	7/9	2/3	6/4	8/5	3/2	3	8	1	5	7	3	ns
Bcl-2 t	0/0	2/0	20/15	0/0	2/2	12/11	0	4	13	0	2	16	ns

^*^IRS index: 0 – no changes, 1 – low grade, 2 – mild, 3 – high grade, t – tubules, g – glomeruli.

**Table 6:** Donor and recipient characteristics in examined groups (standard and IBT).

	STANDARD	IBT	p
Donor gender	10F/13M		
Donor age (years, mean ± SD)	48 ± 12		
Donor last creatinine (mg/dL)	1.13 ± 0.46		
Donor cause of death	16 cerebrovascular accident, 7 other		
CIT (hours, mean ± SD)	28.6 ± 7.4	27.4 ± 7.5	ns
WIT (minutes, mean ± SD)	23.6 ± 8.1	0	p < 0.0001
Recipient gender	10F/13M	7F/16M	ns
Recipient age (years, mean ± SD)	50 ± 13	52 ± 10	ns
Primary kidney disease:			
*Chronic glomerulonephritis*	10	10	
*Hypertensive nephropathy*	4	6	
*Polycystic renal disease*	1	3	
*Chronic interstitial nephritis*	6	1	
*Other/unknown*	2	3	
Time of dialysis before transplantation (months, mean ± SD)	27.5 ± 19.8	28.0 ± 18.1	ns
Dialysis method (HD/PD)	19/4	20/3	ns
HLA mismatches (mean ± SD)	4.1 ± 1.1	3.9 ± 1.0	ns
Max. PRA >20%	4	2	ns
Last PRA >20%	2	0	ns
Second transplant	4	1	ns
Immunosuppression			
*TAC/MPA/steroids*	16	13	
*CsA/MPA/steroids*	7	9	
*mTORI/TAC/steroids*	1		
*Induction with anti-CD25*	1	2	

^*^HD-hemodialysis, PD-peritoneal dialysis, PRA-panel reactive antibodies, TAC-tacrolimus, MPA- mycophenolate mofetil/sodium, CsA-cyclosporine A, mTORI- mTOR inhibitors.
